# Prediction of response to pemetrexed in non-small-cell lung cancer with immunohistochemical phenotyping based on gene expression profiles

**DOI:** 10.1186/s12885-019-5645-x

**Published:** 2019-05-14

**Authors:** S. Visser, J. Hou, K. Bezemer, L. L. de Vogel, J. P. J. J. Hegmans, B. H. Stricker, S. Philipsen, J. G. J. V. Aerts

**Affiliations:** 1 Department of Pulmonary Medicine, Amphia Hospital, Breda, the Netherlands; 2 Department of Pulmonary Medicine, Erasmus MC Cancer Institute, P.O. Box 2040, 3000 CA Rotterdam, The Netherlands; 3 Department of Epidemiology, Erasmus MC, Rotterdam, the Netherlands; 4 Department of Cell Biology, Erasmus MC, Rotterdam, the Netherlands; 5 Department of Pathology, Erasmus MC, Rotterdam, the Netherlands

**Keywords:** NSCLC, Pemetrexed, Response prediction, Gene-expression profiling, Immunohistochemistry

## Abstract

**Background:**

Palliative pemetrexed-based chemotherapy remains a standard of care treatment for the majority of patients with advanced non-squamous non-small-cell lung cancer (NSCLC). Currently, no predictive markers for pemetrexed treatment are available.

**Methods:**

Resected tumour samples from pemetrexed-naïve NSCLC patients were collected. Gene expression profiling with respect to predicted sensitivity to pemetrexed classified predicted responders (60%) and non-responders (40%) based on differentially expressed genes encoding for pemetrexed target enzymes. Genes showing a strong correlation with these target genes were selected for measurement of corresponding protein expressions by immunohistochemical (IHC) staining. A semi-quantitative IHC scoring method was applied to construct a prediction model for response to pemetrexed. A retrospective cohort of patients with advanced NSCLC treated with first-line pemetrexed-based chemotherapy was used for external validation.

**Results:**

From ninety-one patients resected tumour samples were collected. The majority of patients had early or locally advanced NSCLC (96.3%). Gene expression profiling revealed five markers, which mRNA levels strongly correlated to pemetrexed target genes mRNA levels: TPX2, CPA3, EZH2, MCM2 and TOP2A. Of 63 (69%) patients IHC staining scores of these markers were obtained, which significantly differed between predicted non-responders and responders (*P* < 0.05). The optimized prediction model included EZH2 (OR = 0.56, 95% CI 0.35–0.90) and TPX2 (OR = 0.55, 95% CI 0.30–1.01). The model had a sensitivity of 86.8%, specificity of 63.6% and showed a good ability to distinct between responders and non-responders (C-index 0.86).

In the external study population (*N* = 23) the majority of patients had metastatic NSCLC (95.7%). Partial response (PR) was established in 26.1%. The sensitivity decreased drastically to 33.3%, with a specificity of 82.4% and a C-index of 0.73.

**Conclusions:**

Using external validation this prediction model with IHC staining of target enzyme correlated markers showed a good discrimination, but lacked sensitivity. The role of IHC markers as response predictors for pemetrexed in clinical practice remains questionable.

**Electronic supplementary material:**

The online version of this article (10.1186/s12885-019-5645-x) contains supplementary material, which is available to authorized users.

## Background

In the management of advanced non-small cell lung cancer (NSCLC) systemic treatment options are rapidly expanding with the increasing use of molecular targeted agents and immunotherapy [1–4]. One of the most important therapeutic advances has been the identification of predictive molecular markers to guide patient selection for frontline treatment with these agents, like sensitizing mutations within the EGFR gene to EGFR tyrosine kinase inhibitors and protein PD-L1 overexpression to anti PD-(L)1 checkpoint inhibitors [1, 2, 5]. Despite the changing treatment landscape with increasing use of molecular targeted agents and immunotherapy, pemetrexed-based chemotherapy is still widely used as standard treatment in patients with advanced non-squamous non-small-cell lung cancer [6, 7]. Unfortunately, to date useful biomarkers predicting response to this treatment regimen are lacking.

Pemetrexed treatment shows a variable clinical efficacy apparently dependent on the histologic subtype of lung cancer. Clinical trials demonstrated efficacy of pemetrexed in nonsquamous NSCLC while efficacy was worse in squamous NSCLC and small-cell lung cancer [8–10]. However, tumour response to pemetrexed also differs significantly between patients with similar histology [9, 11]. In patients treated with pemetrexed monotherapy, the response rate to pemetrexed was evidently different between histological subtypes but low in both patients with squamous and non-squamous NSCLC (2.8% vs. 11.5%) [10]. In this study pemetrexed was administered as second-line treatment and patients with poor ECOG performance score were included. Although the response rate to pemetrexed was significantly higher in patients with non-squamous versus squamous NSCLC in the first-line pivotal trial [9], still more than 20% of patients with squamous NSCLC had a response to pemetrexed while the response rate was merely ~ 30% in patients with non-squamous histology [11]. These findings highlight the need for predictive molecular markers for pemetrexed-based treatment.

The main determinant of pemetrexed responsiveness is thought to be the level of expression of thymidylate synthase (TS), the primary intracellular target enzyme of pemetrexed [12, 13]. Overexpression of TS mRNA has been correlated with reduced sensitivity to pemetrexed in vitro, [14–17] and with worse clinical outcomes in patients treated with pemetrexed [18]. Moreover, the abundance of TS expression is higher in squamous cell NSCLC than in other histologic subtypes, [19, 20] which constitutes the biological hypothesis behind the superior efficacy of pemetrexed in non-squamous NSCLC. However, in clinical practice the relationship between protein expression levels of TS, measured by immunohistochemistry (IHC) methods, and the clinical efficacy of pemetrexed remains controversial [21–25].

Our study group earlier presented an approach to implement a more refined molecular classification of NSCLC subtypes based on gene expression profiles independent of histology [26]. Furthermore, response to pemetrexed was predicted based on expression of genes encoding different pemetrexed target enzymes including but not limited to TS, and expression signatures of correlated genes were identified. In the current study, we explore whether these differential gene expression profiles between responders and non-responders can be used to define a prediction model based on IHC scores of selected molecular markers.

## Methods

### Training cohort

Tumour samples from pemetrexed-naïve, NSCLC patients who had undergone curative surgical resection at the Erasmus Medical Center (Rotterdam) between 1992 and 2004 were used. A detailed description of tissue collection, microarray preparation and data processing, the derivation of a gene-expression based predictive algorithm for tumour response and the identification of pemetrexed resistance-associated genes has been previously described [26]. In short, the predictive algorithm predicted tumour response based on the expression difference between internal reference genes and pemetrexed target genes TS, dihydrofolate reductase (DHFR) and glycinamide ribonucleotide formyltransferase (GARFT). Using percentile-rank based target gene expression levels relative to the internal reference genes, patients were stratified as predicted responders (±60%) and non-responders (±40%). Subsequently, significance analysis of microarray identified differentially expressed genes between these classified groups [27]. A minimized signature containing 25 genes performed optimally in predicting pemetrexed response (Additional file 1: Table S1). For the current study, we selected molecular markers from this signature if they showed a strong correlation with the gene expression of TS and if IHC stainings for these markers were commercially available. Written informed consent was obtained from all these patients. The study was conducted in accordance with the REMARK guidelines [28].

### Validation cohort

In order to externally validate the model, we obtained formalin-fixed paraffin-embedded pre-treatment biopsies of a retrospective cohort of patients newly diagnosed with advanced stage (IIIB/IV) NSCLC in a large teaching hospital (Amphia hospital, Breda, the Netherlands) between January 2007 and December 2010. Patients were eligible for enrolment if they had received ≥2 cycles of platinum-combined pemetrexed chemotherapy as first-line treatment. Medical charts and radiological imaging data were reviewed to collect information regarding socio demographics, tumour histology, ECOG performance status, treatment and observed tumour response (RECIST 1.1). Patients with early stage (IA-IIB) or locally advanced (IIIA) disease, (neo)adjuvant chemotherapy, combination treatment with bevacizumab and without tissue samples from primary tumour or (lymph node) metastasis were excluded.

### Tissue microarray analysis and immunohistochemistry

The tissue microarrays (TMAs) were composed of 68 of the 91 tumour tissues, in triplicate, from the Erasmus MC patient cohort used for the expression microarray analyses. TMA blocks containing 0.6 mm cores of formalin-fixed paraffin-embedded tumours were cut and antigen retrieval was performed by a 20-min incubation at 95 °C using Trisethylenediaminetetraacetic acid buffer (Klinipath, Duiven, The Netherlands). Subsequently, TMAs were stained with primary antihuman antibodies of the selected candidate markers: EZH2, TOP2A, TPX2, MCM2 and CPA3 (Additional file 2: Table S2). For each TMA multicontrol stainings were performed using a combination of tissues (liver, pancreas, tonsil, colon and appendix). The slides were stained and processed in the Ventana Benchmark ULTRA strainers, using DAB as substrate and Hematoxylin as counterstain. Tumour tissues from the validation group were equally handled, except that the tumour samples were cut in 0.4 mm instead of 0.6 mm cores for TMA blocks.

### Immunohistochemical staining score

A semi-quantitative scoring method was applied to classify the intensity and quantity of IHC staining of candidate markers. The quantity score was defined as: 1: 0–30%; 2: 30–60%; 3: 60–100%. The intensity score was defined as: 0 (negative), no appreciable staining in the tumour cells; 1 (weak), barely detectable cytoplasmic/membranous or nuclear staining of tumour cells; 2 (moderate), readily appreciable staining of tumour cytoplasm/nucleus; 3 (strong), strong staining obscuring nucleus/cytoplasm of tumour cells. Multiplying quantity and intensity score yielded a total score with a range between 0 and 9. TMAs from the training group were evaluated and scored for protein expression simultaneously by K.B. and J.P.H. Samples were individually discussed until consensus was reached. For the validation group, TMA evaluation and protein staining quantification were performed independently by K.B. and S.V.

### Statistical analyses

Sociodemographic and disease- and treatment-related variables were described for all patients who were included in this study and were compared between the training and validation group. We used the independent-samples *t*-test and the *χ2*-test or Fisher’s exact test for continuous and categorical variables respectively. Degree of agreement on quantity and intensity scores of the different IHC stainings was evaluated using weighted linear Cohen’s kappa scores (휅) in the validation group. Degree of agreement was determined according to widely used scale described by Landis and Koch [29]. As IHC staining scores from the training group were obtained by discussion, and thus not independently, no interobserver agreement could be calculated.

Pearson correlation coefficients (*ρ*) were calculated between gene expression of the candidate markers and TS in the training group and subsequently between the gene expression of those markers and their associated protein expressions. Using the described prediction algorithm of response to pemetrexed, patients from the training group were divided in predicted responders and non-responders. In the training group, we compared gene and protein expression from selected molecular markers between predicted responders and non-responders.

Logistic regression with dependent variable predicted tumour response by gene expression signature was applied to the training cohort to build a prediction model with the IHC staining scores of selected molecular markers as independent variables. Optimized model derivation was performed using purposeful selection by stepwise in- and exclusion of molecular markers [30]. Univariable logistic regression identified molecular markers associated with predicted tumour response. We specified a priori that molecular markers with *p* < 0.2 on univariable analysis would be candidate variables for multivariable logistic regression model. In the iterative process of variable selection, variables were removed from the model if they were non-significant and not a confounder. We used backward selection with a *p*-value < 0.05 and/or change of effect size of (an)other included variable(s) > 20% to remain in the model. The fit of a reduced model versus full model was compared with the likelihood ratio test (LRT), following a chi-squared distribution. Subsequently, the model was externally validated in the validation cohort. In both cohorts, the model performance of the derived model was assessed by examining the predictive classification accuracy and discriminatory ability (C-index). A C-index of 1.0 would indicate perfect discrimination, whereas a C-index of 0.5 indicates total absence of discrimination. All statistical analyses were performed with the use of SPSS, version 24.0 (IBM Corporation, Armonk, NY).

## Results

**Fig. 1:**
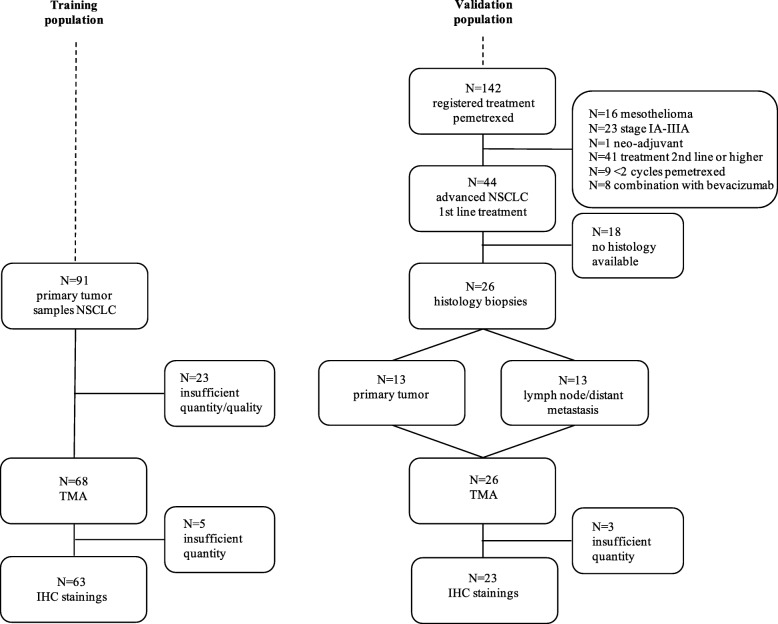
Flowchart of training and validation population. Abbreviations: NSCLC, non-small-cell lung cancer; TMA, tissue micro array; IHC, immunohistochemical

The selection of patients in both training and validation cohort is depicted in Fig. 1. Of the 91 surgically resected samples of the primary tumour, 68 (74.7%) samples were suitable for further processing into TMAs. Since samples of five patients could not be used due to insufficient TMA material, the training cohort ultimately consisted of 63 patients whose samples were prepared with additional IHC stainings. For validation, 44 of 142 (31%) patients who received pemetrexed had advanced stage NSCLC treated with ≥2 cycles first-line treatment with platinum-combined pemetrexed, excluding combinations with bevacizumab. Of these patients, 18 (40.9%) were excluded from further analysis because their diagnosis was cytology-based and no histologic biopsy was obtained. Histology samples were retrieved from primary tumour (*N* = 13) or lymph nodes or distant metastases (*N* = 13). Three patients had insufficient tumour material available for additional IHC staining and therefore the validation cohort consisted of 23 patients.

### Patient characteristics

Patient and treatment characteristics are shown in Table 1. In the training cohort 46 patients (73.0%) were male compared to 10 (43.5%) in the validation cohort. Eighteen patients (28.6%) had squamous NSCLC in the training cohort while in the validation cohort only patients were included with non-squamous histology. The majority of patients (95.2%) in the training group had early stage NSCLC opposed to all patients with advanced disease stage in the validation group. No data were available with regard to the ECOG performance score of the patients in the training cohort. In the validation cohort, 21.7% of the patients had a performance score of 2. Corresponding to the differences in disease stage between the cohorts, all patients in the training cohort underwent surgical resection in contrast to palliative pemetrexed-based chemotherapy in the validation cohort. Patients in the training cohort had worse overall survival compared to the validation cohort (4.5 months vs. 28 months).

**Table 1:** Characteristics of patients in the training population and the validation population

	Training cohort (*N* = 63)	Validation cohort (*N* = 23)
Age, mean (SD)	61.9 (±10.7)	58.7 (±8.7)
Gender, male	46 (73.0)	10 (43.5)
Smoking status		
Never smoker	1 (1.6)	1 (4.3)
Ever smoker	31 (49.2)	21 (91.3)
Unknown	31 (49.2)	1 (4.3)
ECOG performance score		
0 or 1		17 (73.9)
2		5 (21.7)
Unknown	63 (100)	1 (4.3)
Histology		
ADC	18 (28.6)	21 (91.3)
LCC	24 (38.1)	2 (8.7)
SCC	15 (23.8)	
Other	6 (9.5)	
Tumor stage		
IA-IIB	56 (88.9)	
IIIA	4 (6.3)	
IIIB		1 (4.3)
IV	3 (4.8)	22 (95.7)
Treatment		
Surgery	63 (100)	
CISPEM		18 (78.3)
CARPEM		5 (21.7)
No. cycles chemotherapy, median (IQR)		3 (3–4)
Treatment effect		
PR		6 (26.1)
SD		7 (30.4)
PD		10 (43.5)
OS, median (IQR)	28.0 (10.0–67.6)	4.5 (3.2–7.3)

Data are expressed as numbers (%) unless stated otherwise. Abbreviations: *SD* standard deviation, *ADC* adenocarcinoma, *LCC* large cell carcinoma, *SCC* squamous cell carcinoma, *CISPEM* cisplatin combined with pemetrexed, *CARPEM* carboplatin combined with pemetrexed, *OS* overall survival, *IQR* interquartile range, *PR* partial response, *SD* stable disease, *PD* progressive disease

### Selection molecular markers

Of the 25-genes containing optimized gene expression signature predicting response to pemetrexed in the training group, five molecular markers were selected based on their correlation with the gene expression level of target genes (TS, DHFR, GARFT) and the commercial availability of corresponding IHC stainings: Enhancer of zeste homolog 2 (EZH2), Topoisomerase II Alpha (TOP2A), Microtubule Nucleation Factor (TPX2), Carboxypeptidase A3 (CPA3) and Minichromosome Maintenance Complex Component 2 (MCM2). All markers showed a positive correlation to the mRNA level of TS (EZH2, *ρ* = 0.732; MCM2, *ρ* = 0.804; TOP2A, *ρ* = 0.814; TPX2, *ρ* = 0.825), except for CPA3 which was negatively correlated (*ρ* = − 0.467) (Additional file 4: Figure S1). The correlation of gene mRNA level with their corresponding IHC staining score had a range between 0.303 (CPA3) and 0.578 (EZH2) (Additional file 5: Figure S2).

The IHC stainings of the same markers were applied to the TMAs of the samples of patients in the validation cohort. The strength of agreement between the observers with regard to the intensity score ranged between 휅 = 0.515 (CPA3) and 휅 = 1 (MCM2), and with regard to the quantity score between 휅 = 0.547 (TPX2) and 휅 = 0.851 (CPA3). Weighted kappa values of IHC staining scores are outlined in Additional file 3: Table S3.

**Fig. 2:**
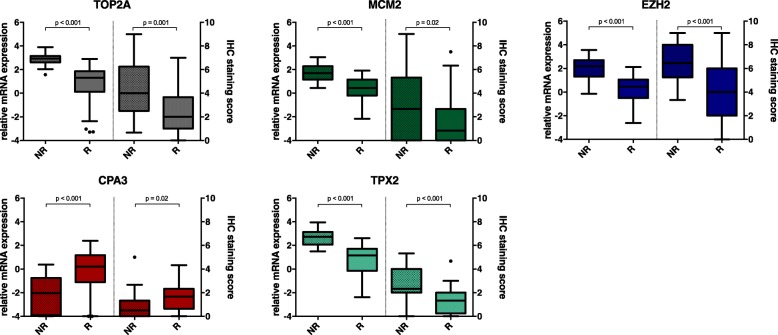
Boxplots of gene and protein expression levels of selected markers in predicted responders and non-responders to pemetrexed in the training group. Boxplots (Tukey) representing medians and interquartile ranges of relative mRNA expression and IHC staining scores respectively. Whiskers represent minimum and maximum 1.5 interquartile range and dots are outliers. Abbreviations: IHC, immunohistochemical; EZH2, Enhancer of zeste homolog; TOP2A, Topoisomerase II; TPX2, Microtubule Nucleation Factor; CPA3, Carboxypeptidase A3; MCM2, Minichromosome Maintenance Complex Component 2. Abbreviations: IHC, immunohistochemical; EZH2, Enhancer of zeste homolog; TOP2A, Topoisomerase II Alpha; TPX2, Microtubule Nucleation Factor; CPA3, Carboxypeptidase A3; MCM2, Minichromosome Maintenance Complex Component 2

Of all selected markers, both mRNA levels and IHC staining scores were significantly higher in predicted non-responders than responders in the training group, except for CPA which mRNA level and IHC staining score was significantly lower in non-responders compared to responders. These results are depicted in Fig. 2.

### Model derivation

The model coefficients and odds ratios (OR) with corresponding 95% confidence intervals (CI) of the prediction model with dependent variable tumour response to pemetrexed using univariable and multivariable logistic regression analyses are presented in Table 2. Univariable analyses of the relationship between the IHC staining scores of the selected markers and the gene expression based predicted tumour response to pemetrexed were performed using the training cohort. Higher IHC staining scores of all markers were significantly associated with worse predicted tumour response, except for CPA3 which repeatedly showed the reverse association compared to the other markers. Using multivariable analysis, only a higher IHC staining score of EZH2 was significantly related with a worse predicted tumour response to pemetrexed (OR 0.56, 95% CI 0.35–0.90; *P* = 0.015). The staining scores of all other markers failed to demonstrate a significant association on multivariable analysis. Although IHC staining score of TPX2 was not significantly associated with tumour response in the multivariable model (OR 0.55, 95% CI 0.30–1.01; *P* = 0.054), this variable was still included in the final optimized prediction model. Removal of this variable led to a significantly reduced model fit (*P* < 0.001).

**Table 2:** Prediction model derivation to predict tumor response using IHC staining scores of selected molecular markers in training group (*N* = 63)

	Univariable analysis		Optimized model	
	Odds ratio (95% CI)	*P*-value	Odds ratio (95% CI)	*P*-value
IHC score CPA3	1.82 (1.08–3.07)	0.025		
IHC score EHZ2	0.47 (0.30–0.71)	< 0.001	0.56 (0.35–0.90)	0.015
IHC score TPX2	0.43 (0.26–0.70)	0.001	0.55 (0.30–1.01)	0.054*
IHC score MCM2	0.75 (0.0.59–0.96)	0.022		
IHC score TOP2a	0.67 (0.51–0.88)	0.003		

*Model fit was significantly worse (based on difference − 2 Log Likelihood) if TPX2 was excluded. Abbreviations: *CI* confidence interval, *EZH2* Enhancer of zeste homolog, *TOP2A* Topoisomerase II Alpha, *TPX2* Microtubule Nucleation Factor, *CPA3* Carboxypeptidase A3, *MCM2* Minichromosome Maintenance Complex Component 2

### Model performance and validation

In Table 3 the different test characteristics describing the performance of the model in the training and validation cohort are shown. In the training cohort, 38 patients were predicted responders (63.3%) by gene expression profiling and 86.8% (33 of 38) were correctly classified responders by the prediction model (sensitivity 86.8%, 95% CI 71.9–95.6). Fourteen patients were predicted non-responders (36.7%) by gene expression profiling while 63.6% (14 of 22) were correctly classified as non-responders by the model (specificity 63.6%, 95% CI 40.7–82.8).

**Table 3:** Conditional and post-test probability performance of the IHC based prediction model in the training and validation cohort

	Training cohort	Validation cohort Responder: PR	Validation cohort Responder: PR + SD
Prevalence (responder)	63.3 (49.9–75.4)	26.1 (10.2–48.4)	56.5 (34.5–76.8)
Sensitivity	86.8 (71.9–95.6)	33.3 (4.3–77.7)	15.4 (1.9–45.5)
Specificity	63.6 (40.7–82.8)	82.4 (56.6–96-2)	70.0 (34.6–93.3)
LR+ (weighted by prevalence)	2.39 (1.36–4.21)	1.89 (0.41–8.71)	0.51 (0.10–2.51)
LR- (weighted by prevalence)	0.21 (0.09–0.50)	0.81 (0.44–1.49)	1.21 (0.76–1.93)
PPV	80.5 (70.1–87.9)	40.0 (12.6–75.5)	40.0 (12.0–76.5)
NPV	73.7 (53.8–87.1)	77.8 (65.6–86.5)	38.9 (28.5–50.4)

Data are expressed as percentages, except LR+ and LR- (odds), with 95% confidence intervals. Abbreviations: *LR+* positive likelihood ratio, *LR-* negative likelihood ratio, *PPV* positive predictive value, *NPV* negative predictive value

In the validation cohort, the same classification by the prediction model was applied, however an actual tumour response was obtained as these patients were treated with pemetrexed. The response rate was 26.1% and therefore the prevalence of response was substantially lower than in the training cohort. Of the six patients who experienced a partial response, two patients were correctly classified by the prediction model resulting in a sensitivity of 33.3% (95% CI 4.3–77.7). Herewith, the sensitivity in this cohort is significantly worse than the sensitivity in the training cohort (Fisher’s exact test, *P* = 0.011). The positive predictive value (PPV) also decreased substantially. If we classified both patients with a partial response and stable disease as responders (56.5%), performance characteristics of the model declined dramatically (Table 3).

**Fig. 3:**
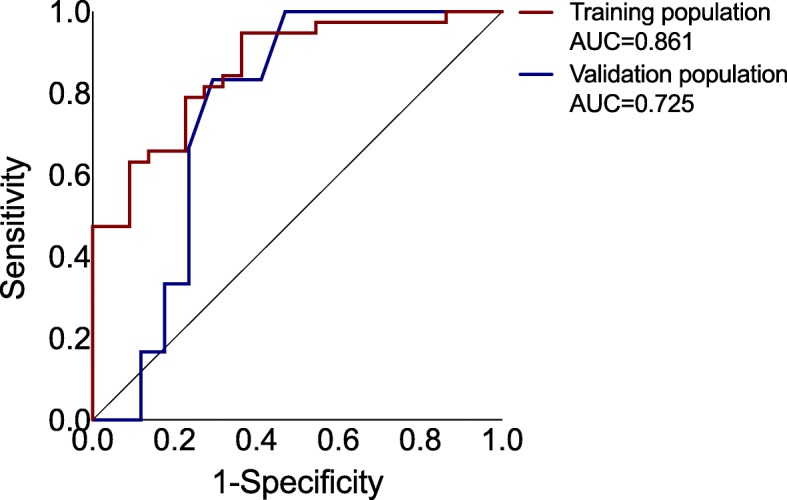
Receiver operating characteristic curve showing model performance of two-protein expression prediction model in training and validation population. Diagonal line reflects total absence of discrimination (AUC = 0.5). Abbreviations: AUC, area under the curve

The ROC curve showed a C-index of 0.86 (95% CI 0.77–0.96) in the training cohort, representing a good discriminatory performance. The C-index decreased to 0.73 (95% CI 0.52–0.93) if the prediction model was applied to the validation cohort (Fig. 3).

## Discussion

Currently, the profit of and need for molecular markers to select therapy for individual patients is increasingly recognized. The last several years, treatment of advanced NSCLC has become obviously more complex and therefore tools to choose therapies that are most likely to benefit patients are required. Indeed the registration of new therapeutic agents as frontline therapy is accompanied by selective markers. For patients with EGFR mutation-positive, ALK rearrangement-positive or ROS1 rearrangement positive tumours first-line molecular targeted tyrosine kinase inhibitors are recommended, while patients with high PD-L1 expression in the tumour are suitable for pembrolizumab first-line treatment [6]. Despite the wide implementation of molecular markers, the administration of chemotherapy in NSCLC patients is still solely based on histology even though its capacity to predict response has been proved to be suboptimal.

In an earlier study, we demonstrated an approach to predict response to pemetrexed based on the use of gene expression profiles [26]. Samples of different histological subtypes including squamous NSCLC were used. Prediction of response to pemetrexed based on gene expression profiling of its target enzymes, failed to show the expected disadvantage for the squamous cell histological subtype and these results therefore challenge the restricted use of pemetrexed in non-squamous NSCLC. In the present study, we developed a prediction model using immunohistochemistry scores of selected candidate genes (EZH2 and TPX2) from the gene expression signature predicting pemetrexed response. In the training group, the use of the model resulted in good performance characteristics of the model in the sense of a high sensitivity and PPV. The model also showed the ability to discriminate well between responders and non-responders. Unfortunately, the results of the obtained model could not be validated when applied to an external cohort of patients treated with pemetrexed.

Although it is hypothesized that superior efficacy of pemetrexed in non-squamous over squamous NSCLC is related to the level of TS expression, multiple clinical studies failed to demonstrate the association between TS protein expression and clinical outcomes [22, 25]. This can be ascribed firstly to the fact that current semi-quantitative IHC methods might lack sensitivity required to measure protein expression of TS opposed to quantitative analysis of mRNA expression. And secondly, the biological significance of TS for pemetrexed responsiveness might be of less importance than other molecular processes, e.g. gene expression or amplification of other (target) genes. To overcome these limitations in the present study, we carefully selected markers related to mRNA gene expression of TS but also to other target enzymes DHFR and GARFT.

In the literature, both EZH2 and TPX2 have been previously linked to survival in NSCLC. TPX2 is involved in key steps during mitotic events and increased expression has been associated with poor overall survival in NSCLC [31, 32]. EZH2 epigenetically silences multiple genes involved in cell differentiation, growth and invasion. It is often overexpressed in NSCLC promoting cancer progression and a more aggressive tumour behaviour [33, 34]. Downregulation of EZH2 has been associated with higher expression of oestrogen receptor and increased sensitivity to tamoxifen in advanced breast cancer patients [35]. Similarly, one can speculate that EZH2 might change the expression of genes related to responsiveness of pemetrexed through its ability to silence other genes. Although lack of a control arm precludes discrimination between a prognostic or predictive factor, we purposefully focused on radiological response rather than survival.

It is crucial to predict treatment effects for individual patients in order to avoid unnecessary toxicities and to offer alternative treatment options, with few false positives [36]. The clinical value of the derived model is probably limited as the sensitivity was poor in the validation group. Moreover, the low PPV makes the classifier not useful for clinical decision making, as many patients who are predicted responders will then actually undergo potentially harmful treatment with low chance of tumour response. The failure of the prediction model to adequately perform in the external patient population might be ascribed to an insufficient sensitivity of used IHC assays to measure significant differences in protein expression or a discrepancy between protein expression and protein activity. Additionally, spatiotemporal heterogeneity might have led to different intrinsic tumour properties in the validation group as these patients had advanced disease and in half of the cases tumour samples were obtained from lymph node or distant metastases. Finally, other factors might influence pemetrexed activity such as cell transport and intracellular formation of polyglutamate metabolites [37]. Our study was limited in the number of patients included, especially in the external validation.

cohort. We recognize that the differences in histology between the training and validation cohort is a major shortcoming. It was impossible to include patients with non-squamous NSCLC to the validation cohort, as selection was treatment-based and pemetrexed is only recommended in patients with nonsquamous NSCLC. For ALK and ROS1-rearrangement positive adenocarcinoma patients might experience more benefit to pemetrexed-based chemotherapy [38, 39], molecular characteristics would have been desirable. Unfortunately, those data were not available in our cohort, but the high number of smokers profoundly reduces the chance of rearrangements. Although response rates were in accordance with the literature, patients in the validation cohort experienced a very poor median overall survival of only 4.5 months. This can be partially explained by the presence of a substantial group of patients (> 20%) with a poor ECOG performance score and suboptimal treatment with carboplatin instead of cisplatin combination. Whether these patients appropriately represent the population with advanced NSCLC is therefore highly questionable and we cannot exclude their genetic profile to be different. However, given the results we do not expect that expanding the number of samples will lead to a clinically useful biomarker.

## Conclusion

There remains an unmet need to identify biomarkers to select patients for standard pemetrexed-based treatment. Prediction of pemetrexed responsiveness with IHC stainings of markers correlated to TS and other target enzymes could not be validated using external validation. Future research focusing on metabolomics, pharmacokinetics and pharmacogenetics might offer new insights into tailoring therapy. Until a well-validated biomarker is identified, histology should remain the standard to select advanced NSCLC patients eligible for treatment with pemetrexed.

## Additional files

Additional file 1: **Table S1.** Minimized signature for prediction of pemetrexed response. (DOCX 66 kb)

Additional file 2: **Table S2.** Antibodies used for immunohistochemical analyses. (DOCX 33 kb)

Additional file 3: **Table S3.** Interobserver agreement of the IHC staining score with regard to tumour quantity and intensity of staining. (DOCX 39 kb)

Additional file 4: **Figure S1.** Scatter plots of gene expression levels of selected molecular markers and TS gene expression. Dot plots showing correlations between relative mRNA expression of TS and mRNA expression of TOP2A, MCM2, EZH2, CPA3, TPX2. Abbreviations: IHC, immunohistochemical; EZH2, Enhancer of zeste homolog; TOP2A, Topoisomerase II; TPX2, Microtubule Nucleation Factor; CPA3, Carboxypeptidase A3; MCM2, Minichromosome Maintenance Complex Component 2. (EPS 180 kb)

Additional file 5: **Figure S2.** Scatter plots of gene expression levels of selected molecular markers with their protein expression level and the associated correlations. Dot plots showing correlations between relative mRNA expression and IHC staining score of TOP2A, MCM2, EZH2, CPA3, TPX2. Abbreviations: IHC, immunohistochemical; EZH2, Enhancer of zeste homolog; TOP2A, Topoisomerase II; TPX2, Microtubule Nucleation Factor; CPA3, Carboxypeptidase A3; MCM2, Minichromosome Maintenance Complex Component 2. (EPS 176 kb)

## Abbreviations

CI: Confidence interval
CPA3: Carboxypeptidase A3
DHFR: Dihydrofolate reductase
EZH2: Enhancer of zeste homolog 2
GARFT: Glycinamide ribonucleotide formyltransferase
IHC: Immunohistochemical
LRT: Likelihood ratio test
MCM2: Minichromosome Maintenance Complex Component 2
NSCLC: Non-small-cell lung cancer
OR: Odds ratio
PPV: Positive predictive value
TMA: Tissue microarrays
TOP2A: Topoisomerase II Alpha
TPX2: Microtubule Nucleation Factor
TS: Thymidylate synthase
