# Supplementary material for: Prediction of response to pemetrexed in non-small-cell lung cancer with immunohistochemical phenotyping based on gene expression profiles
Source: BMC Cancer. 2019 May 14;19:440. doi: 10.1186/s12885-019-5645-x (PMC6515672; doi:10.1186/s12885-019-5645-x)
Supplement: Supplementary file 1 — Table S1. Minimized signature for prediction of pemetrexed response. (DOCX 66 kb) [file 12885_2019_5645_MOESM1_ESM.docx]

| **Table S1 \|** Minimized signature for prediction of pemetrexed response | | | | |
| --- | --- | --- | --- | --- |
| **ProbesetID** | **Gene Symbol** | **NR : R Ratio** | **NR Mean** | **R Mean** |
| 1554696_s_at | TYMS | 3.79 | 1.11 | -0.77 |
| 202589_at | TYMS | 3.70 | 1.15 | -0.83 |
| 202954_at | UBE2C | 3.19 | 1.04 | -0.79 |
| 201292_at | TOP2A | 3.05 | 1.04 | -0.88 |
| 223381_at | NUF2 | 3.04 | 0.93 | -0.74 |
| 204162_at | NDC80 | 2.84 | 0.87 | -0.73 |
| 207828_s_at | CENPF | 2.79 | 0.97 | -0.80 |
| 210052_s_at | TPX2 | 2.77 | 1.04 | -0.79 |
| 204146_at | RAD51AP1 | 2.74 | 0.86 | -0.64 |
| 222958_s_at | DEPDC1 | 2.73 | 0.80 | -0.67 |
| 218755_at | KIF20A | 2.70 | 0.79 | -0.68 |
| 219918_s_at | ASPM | 2.67 | 0.93 | -0.75 |
| 203358_s_at | EZH2 | 2.65 | 0.87 | -0.70 |
| 201291_s_at | TOP2A | 2.63 | 1.05 | -0.85 |
| 204822_at | TTK | 2.55 | 0.88 | -0.72 |
| 204962_s_at | CENPA | 2.54 | 0.88 | -0.68 |
| 219306_at | KIF15 | 2.53 | 0.73 | -0.55 |
| 202107_s_at | MCM2 | 2.52 | 0.90 | -0.65 |
| 205053_at | PRIM1 | 2.50 | 0.74 | -0.55 |
| 222680_s_at | DTL | 2.48 | 0.83 | -0.67 |
| 218039_at | NUSAP1 | 2.39 | 0.79 | -0.68 |
| 204444_at | KIF11 | 2.35 | 0.69 | -0.57 |
| 204023_at | RFC4 | 2.27 | 0.85 | -0.65 |
| 39248_at | AQP3 | 0.40 | -1.34 | 0.78 |
| 205624_at | CPA3 | 0.35 | -1.18 | 0.75 |
|  |  |  |  |  |
| **NR: predicted non-responder to Pemetrexed** | | |  |  |
| **R: predicted responder to Pemetrexed** | | |  |  |
