# Supplementary material for: Prediction of response to pemetrexed in non-small-cell lung cancer with immunohistochemical phenotyping based on gene expression profiles
Source: BMC Cancer. 2019 May 14;19:440. doi: 10.1186/s12885-019-5645-x (PMC6515672; doi:10.1186/s12885-019-5645-x)
Supplement: Supplementary file 2 — Table S2. Antibodies used for immunohistochemical analyses. (DOCX 33 kb) [file 12885_2019_5645_MOESM2_ESM.docx]

| **Table S2 \|** Antibodies used for immunohistochemical analyses | | | |
| --- | --- | --- | --- |
| **Name** | **Company** | **Titer** | **Antibody type** |
| EZH2 | Leica | 1:200 | Anti-human |
| TOP2A | Leica | 1:40 | Anti-human |
| TPX2 | Sigma | 1:100 | Anti-human |
| MCM2 | Bio Connect | 1:100 | Anti-human |
| CPA3 | Sigma | 1:1000 | Anti-human |
