# Supplementary material for: Prediction of response to pemetrexed in non-small-cell lung cancer with immunohistochemical phenotyping based on gene expression profiles
Source: BMC Cancer. 2019 May 14;19:440. doi: 10.1186/s12885-019-5645-x (PMC6515672; doi:10.1186/s12885-019-5645-x)
Supplement: Supplementary file 3 — Table S3. Interobserver agreement of the IHC staining score with regard to tumour quantity and intensity of staining. (DOCX 39 kb) [file 12885_2019_5645_MOESM3_ESM.docx]

| **Table S3 \|** Interobserver agreement of the IHC staining score with regard to tumor quantity and intensity of staining | | | | |
| --- | --- | --- | --- | --- |
|  | **Tumor quantity  N = 23** | | **Tumor Intensity N = 23** | |
|  | weighted kappa (𝜅) | 95% CI | weighted kappa (𝜅) | 95% CI |
| TPX2 | 0.547 | 0.220-0.873 | 0.577 | 0.025-1.130 |
| CPA3 | 0.851 | 0.684-1.019 | 0.515 | 0.203-0.827 |
| EZH2 | 0.723 | 0.441-1.006 | 0.733 | 0.514-0.951 |
| MCM2 | 0.741 | 0.531-0.951 | 1 |  |
| TOP2A | 0.785 | 0.563-1.006 | 0.708 | 0.336-1.080 |
|  | | | | |
